# How membranes shape plant symbioses: signaling and transport in nodulation and arbuscular mycorrhiza

**DOI:** 10.3389/fpls.2012.00223

**Published:** 2012-10-05

**Authors:** Laure Bapaume, Didier Reinhardt

**Affiliations:** Department of Biology, University of FribourgFribourg, Switzerland

**Keywords:** symbiosis, arbuscule, mycorrhiza, LysM receptor, SYMRK, VAPYRIN, root nodules, rhizobium

## Abstract

As sessile organisms that cannot evade adverse environmental conditions, plants have evolved various adaptive strategies to cope with environmental stresses. One of the most successful adaptations is the formation of symbiotic associations with beneficial microbes. In these mutualistic interactions the partners exchange essential nutrients and improve their resistance to biotic and abiotic stresses. In arbuscular mycorrhiza (AM) and in root nodule symbiosis (RNS), AM fungi and rhizobia, respectively, penetrate roots and accommodate within the cells of the plant host. In these endosymbiotic associations, both partners keep their plasma membranes intact and use them to control the bidirectional exchange of signaling molecules and nutrients. Intracellular accommodation requires the exchange of symbiotic signals and the reprogramming of both interacting partners. This involves fundamental changes at the level of gene expression and of the cytoskeleton, as well as of organelles such as plastids, endoplasmic reticulum (ER), and the central vacuole. Symbiotic cells are highly compartmentalized and have a complex membrane system specialized for the diverse functions in molecular communication and nutrient exchange. Here, we discuss the roles of the different cellular membrane systems and their symbiosis-related proteins in AM and RNS, and we review recent progress in the analysis of membrane proteins involved in endosymbiosis.

## Introduction

In nature, the majority of plants live in association with fungal and/or bacterial symbionts. The most widespread symbiosis in all taxa of extant land plants is arbuscular mycorrhiza (AM). The fossil record and phylogenetic analysis suggest an early origin of AM before the Devonian period, approximately 450 Ma ago (Redecker et al., 2000; Heckman et al., 2001; Kistner and Parniske, 2002). AM occurs between fungi of the phylum *Glomeromycota*, also referred to as AM fungi, and the majority of land plants in almost all ecological niches (Wang and Qiu, 2006), and is thought to be essential for plant survival in harsh environments such as deserts and hot springs (Bunn et al., 2009; Al-Yahya'ei, 2011). Whereas AM fungi can colonize the majority of land plants, root nodule symbiosis (RNS) with bacteria (rhizobia), which has evolved considerably later than AM (Kistner and Parniske, 2002), involves almost exclusively legumes (*Fabaceae*).

AM and RNS are both regulated by a common set of genes that define the common SYM pathway. They encode a receptor kinase localized to the plasma membrane, components of signal transduction to the nucleus, and a nuclear CCaMK (calcium and calmodulin-dependent protein kinase; Parniske, 2008; Oldroyd et al., 2011; Singh and Parniske, 2012).

Upon detection of AM fungal hyphopodia, epidermal cells generate an infection structure, the prepenetration apparatus (PPA) that is essential for infection of epidermal cells (Genre et al., 2005, 2008). At later stages of AM, finely branched hyphal structures, the arbuscules, are formed by AM fungi which serve to increase the surface area for nutrient exchange. In RNS, root hair cells form a curl in which bacteria are entrapped and subsequently guided through an infection thread (IT) toward the root cortex (Fournier et al., 2008). Cortical cells prepare for infection with a pre-infection thread (PIT) before they come into contact with the rhizobia (Van Brussel et al., 1992). Ultimately, in the mature nodules, bacteria differentiate into bacteroids inside the cytoplasm of the host (Jones et al., 2007; Oldroyd et al., 2011).

**Figure 1:**
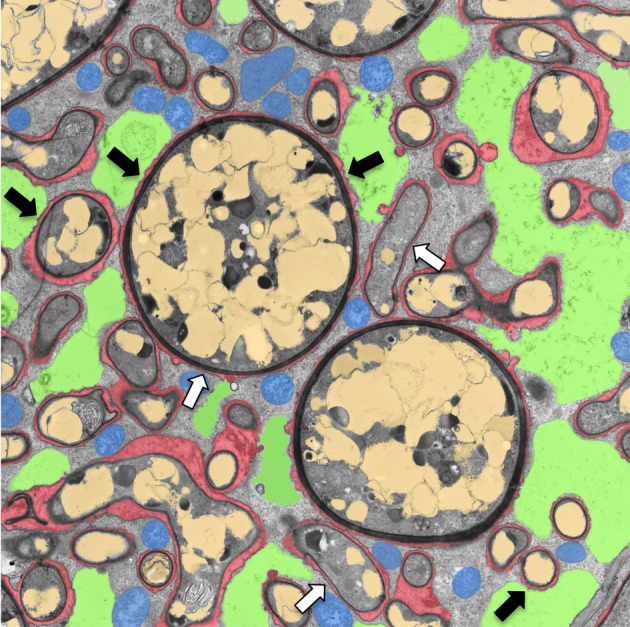
**Transmission electron micrograph of a cortical cell of *P. hybrida* colonized by *G. intraradices* (*Rhizophagus irregularis*).** For clarity, cellular components are pseudocolored as follows: green, fragmented plant vacuole; blue, plant mitochondria and plastids; light brown, fungal vacuoles; red, symbiotic interface. Note the very close contact of the periarbuscular membrane (PAM) with fungal hyphae (white arrows), and the proximity of the tonoplast with the PAM (black arrows).

**Figure 2:**
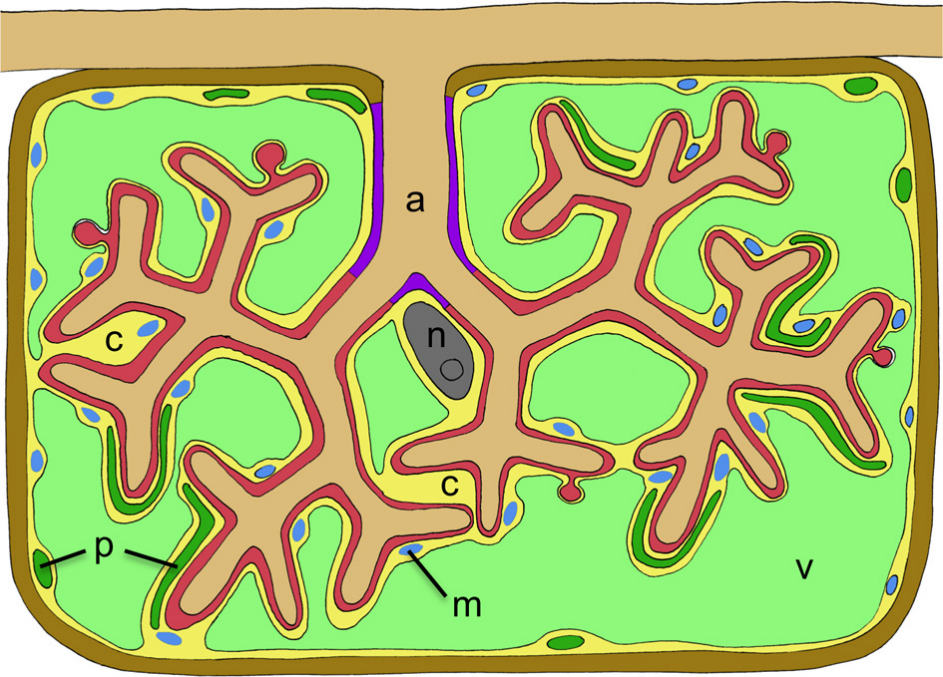
**Schematic representation of a cortex cell with an arbuscule.** The arbuscule takes most of the space that is normally occupied by the central vacuole. Cellular compartments are colored in light green (plant vacuole), dark green (plant plastids), blue (plant mitochondria), yellow (plant cytoplasm), gray (nucleus), red (symbiotic interface), purple (trunc portion of the symbiotic interface), and brown (plant cell wall). The cellular constituents of the host are marked with letters as follows: c, cytoplasm; m, mitochondria; n, nucleus; p, plastids; v, vacuole. The fungal arbuscule is marked as well (a).

The arbuscules and bacteroids are contained within host-derived membranes that represent specialized symbiotic interfaces dedicated to nutrient exchange (Spaink, 1995; Limpens et al., 2005; Parniske, 2008). As a consequence of the large contact area between the host and the endosymbiont, the membrane surface area of host cells (comprising plasma membrane and the membrane around the endosymbiont) increases several-fold during arbuscule formation (Cox and Sanders, 1974), and up to 20-fold in the case of nodule cells filled with nitrogen-fixing bacteria (Verma et al., 1978). Similarly, the endomembrane system undergoes a general expansion since the amount of organelles such as ER, plastids, and mitochondria is amplified (Genre et al., 2005; Lohse et al., 2005; Fournier et al., 2008; Genre et al., 2008; Figures 1 and 2). These adaptations during the transition of a cortical cell to an active symbiotic machinery requires the production of large amounts of new membrane material in the host, and of specialized membrane proteins for symbiotic communication and nutrient exchange.

While the components involved in recognition and signal transduction are expressed constitutively, the machinery required for the functioning of endosymbioses is induced as a consequence of the transcriptional reprogramming of the symbiotic host cells. Many of these genes, which encode among others transporters of various mineral nutrients, are expressed only in symbiotic cells and are therefore likely to play symbiosis-specific roles. In the case of AM, the plant receives nutrients such as phosphorus (P), nitrogen (N), sulfur (S), zinc (Zn), and copper (Cu), which are taken up by the periarbuscular membrane (PAM) in arbuscule-containing cells (Clark and Zeto, 2000; Karandashov and Bucher, 2005; Allen and Shachar-Hill, 2009; Tian et al., 2010; Smith and Smith, 2011), whereas in RNS, the plant is provided with N only (Prell and Poole, 2006). In exchange plants provide carbohydrates (C) to their symbionts (Prell and Poole, 2006; Smith and Smith, 2011). Consistent with a central role of membranes in symbiosis, a large part of the symbiosis-related proteins are localized to membranes. Here, we discuss the different roles of membrane systems in endosymbiosis and we review recent progress in the analysis of symbiosis-related proteins on membranes and their roles in signaling, intracellular accommodation, and nutrient transport.

## Symbiotic signaling

### Flavonoids, strigolactones, nod factors, and myc factors

The rhizosphere is a habitat for a plethora of microbes (Pini et al., 2012). Most of them are neutral commensalists, but some are relevant for plants, either as pathogens or as mutualists. Since it is vital for the plant to react early and adequately, communication in the rhizosphere is crucial for plant survival. Most plant species constitutively release from their roots diffusible signal molecules, strigolactones that stimulate hyphal branching in AM fungi (Akiyama et al., 2005; Besserer et al., 2006), as well as in fungal pathogens (Dor et al., 2011). However, whereas AM fungal metabolism is stimulated by strigolactones (Besserer et al., 2006), the growth of fungal pathogens is inhibited (Dor et al., 2011).

**Figure 3:**
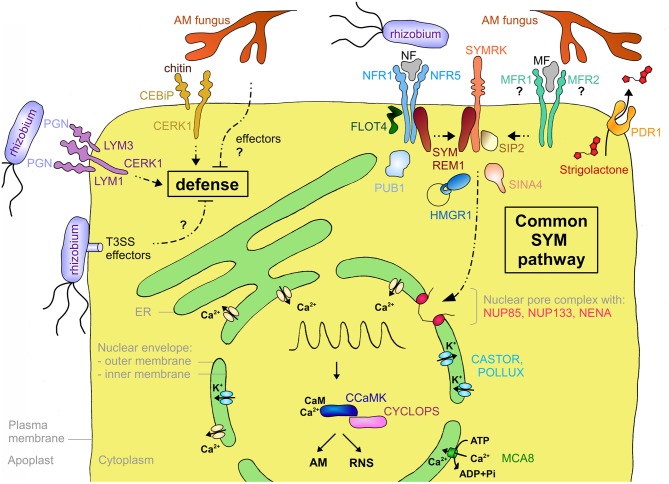
**Schematic representation of a plant cell with the major components involved in symbiotic signaling and defense signaling.** The central vacuole has been omitted for clarity. Solid arrows indicate transport fluxes whereas dashed arrows represent signaling pathways. Receptor complexes involving LysM proteins originate from different plant species. Perception of bacterial peptidoglycan (PGN) is represented by CERK1, LYM1, and LYM3 of *Arabidopsis*. Chitin perception is represented by rice proteins CERK1 and CEBiP. The nod factor receptors (NFR1 and NFR5) are from *L. japonicus*, whereas the elusive nature of the myc factor receptors (MFR1 and MFR2) is shown with question marks. The common SYM pathway is represented by SYMRK, NENA, NUP85, NUP133, CASTOR, POLLUX, CCAMK, and CYCLOPS from *L. japonicus*. The remaining components (MCA8, SIP2, FLOT4, PUB1, SYMREM1, SINA4, and HMGR1) were described in *M. truncatula* or *L. japonicus*, except for PDR1 that was discovered in petunia. See Table A1 and the main text for more information on the respective genes and their function in symbiosis.

Strigolactone is secreted from roots of petunia (*Petunia hybrida*) by the ATP-binding cassette subtype G (ABCG) transporter PDR1 (Kretzschmar et al., 2012; Figure 3). *PDR1* is expressed preferentially during P starvation, a condition that favors AM. PDR1 is localized to the plasma membrane of the subepidermal passage cells, which are the preferred entry point for AM fungi (Sharda and Koide, 2008). Hence, PDR1 may play a role in establishing strigolactone gradients that direct AM fungal hyphae toward suitable points for root penetration (Kretzschmar et al., 2012).

The roots of legumes secrete flavonoids that are perceived as diffusible attractants by rhizobia and that activate them to produce a specific symbiotic signal, the nod factor (NF; Hassan and Mathesius, 2012). NFs are lipochitooligosaccharides (LCOs) that induce early plant responses such as root hair curling (Gough and Cullimore, 2011) and nodule organogenesis.

Only recently, LCO signal molecules similar to NFs were isolated from AM fungi, referred to as myc factors (MF; Maillet et al., 2011), indicating that AM and RNS involve similar symbiotic signals. In view of the obvious similarities in signaling between AM and RNS, it is still a mystery why RNS is characterized by a distinct host-specificity and very narrow host ranges (Wang et al., 2012), whereas AM exhibit a very low degree of specificity, resulting in extremely large host ranges (Smith and Read, 2008).

### LysM receptors

Legumes have dedicated NF receptors (NFRs) that are localized to the plasma membrane and consist of an extracellular domain with two to three lysin motif (LysM) repeats and an intracellular kinase domain (Madsen et al., 2003; Radutoiu et al., 2003; Arrighi et al., 2006; Lohmann et al., 2010). LysM repeats were first identified in bacterial enzymes where they are involved in the binding of peptidoglycans (Buist et al., 2008). LysM-containing receptor-like kinases (LYKs) are plant-specific and occur as families of 5–21 members per species (Zhang et al., 2009). NF perception requires two LysM-containing proteins, which may function as dimers like many eukaryotic receptor systems (Gough and Cullimore, 2011; Gust et al., 2012; Figure 3). In *Lotus japonicus* they are referred to as Nod factor receptor1 (NFR1) and NFR5 (Madsen et al., 2003; Radutoiu et al., 2003), whereas in *Medicago truncatula* they are referred to as LYK3 and Nod factor perception (NFP), respectively (Ben Amor et al., 2003; Smit et al., 2007). Interestingly, the members of one of the subfamilies (including NFR5 and NFP) have a non-functional kinase domain, consistent with the idea that they may form a signaling complex with a second receptor that contains a functional kinase domain (Madsen et al., 2011). Domain swapping experiments between different NFRs and mutation analysis of the extracellular LysM domain support the idea that this part of the receptor (in particular LysM repeat 2) may be involved in the recognition of NFs (Radutoiu et al., 2007; Bensmihen et al., 2011). Indeed, NFR1 and NFR5 were recently shown to bind NF, presumably with their glycosylated extracellular LysM domain (Broghammer et al., 2012).

LCOs have an *N*-acetylglucosamine backbone (Dénarié et al., 1996) that they share with chitin and peptidoglycan (Lovering et al., 2012), the major components of fungal and bacterial cell walls, respectively. Plants have very sensitive receptors for chitin and peptidoglycan oligomers that are structurally related to NFRs (Figure 3). In rice (*Oryza sativa*), two LysM-containing proteins, CEBiP (chitin oligosaccharide elicitor-binding protein) and CERK1 (chitin elicitor receptor kinase1), interact to form a chitin receptor at the plasma membrane (Kaku et al., 2006; Shimizu et al., 2010). In *Arabidopsis*, CERK1 which contributes to resistance against fungal pathogens, and LYM2 (LysM-containing protein2), a close homolog of CEBiP, can bind chitin (Miya et al., 2007; Wan et al., 2008; Petutschnig et al., 2010). However, recent mutant analysis suggests that despite its chitin-binding activity LYM2 is dispensable for chitin signaling (Shinya et al., 2012). Indeed, CERK1 alone, in particular its LysM repeat 2, can bind chitin oligomers and dimerize to form a functional receptor (Liu et al., 2012). Nevertheless, another LysM protein, LYK4, contributes to chitin signaling (Wan et al., 2012). Interestingly, CERK1 of *Arabidopsis* could also form a trimeric receptor complex with LYM1 and LYM3 that recognizes bacterial peptidoglycan. The binding activity is attributed to LYM1 and LYM3, whereas CERK1 appears to be responsible for subsequent defense signaling (Gimenez-Ibanez et al., 2009; Willmann et al., 2011). These results suggest that in general LysM-containing receptors may be formed by combinatorial oligomerization of different LYKs and LYMs (Figure 3). Notably, despite its inability to engage in symbiosis, *Arabidopsis* can perceive NFs at nanomolar concentrations (Khan et al., 2011), indicating that chitin or peptidoglycan receptors may have an affinity for LCOs. The fact that AM-competent plants such as rice (see above), and *M. truncatula* (Fliegmann et al., 2011) have chitin receptors raises the question how AM fungi escape defense response (see below).

While the NFRs of legumes recognize only one or few NFs, thereby limiting the host range in RNS (Wang et al., 2012), an NFP homolog of the non-legume *Parasponia andersonii* (*Cannabaceae*) serves as a common receptor in AM and RNS (Op Den Camp et al., 2011), suggesting that in this case the receptor can recognize different NFs and MFs. These results indicate that AM and RNS may have originally depended on the same receptor(s), which later diversified to produce functionally separate receptors for MFs and NFs in legumes. The functional characterization of further MF receptors (MFRs) from non-legume species will help understand the evolution and function of the LYKs.

### SYMRK

A central component of symbiotic signaling is the symbiosis receptor-like kinase SYMRK that is essential for both AM and RNS (Figure 3). *SYMRK* was initially identified in *Medicago sativa* and *L. japonicus* (Endre et al., 2002; Stracke et al., 2002) but later was found to be conserved in most angiosperms. *SYMRKs* from different symbiosis-competent species in different families can complement each other indicating that SYMRK is functionally conserved and does not contribute to host specificity in RNS (Gherbi et al., 2008; Markmann et al., 2008). Indeed, SYMRK is considered to be the first component of the common SYM pathway which presumably integrates intermediary signals resulting from perception of MF and NF at the plasma membrane (Parniske, 2008).

In addition to its role in AM and in RNS of legumes, SYMRK is also involved in the actinorrhizal nodule symbiosis of *Casuarina glauca* (Fagales) and *Datisca glomerata* (Cucurbitales) with actinobacteria of the genus *Frankia* (Gherbi et al., 2008; Markmann et al., 2008). Hence, SYMRK can be considered the central symbiotic entry point of endosymbioses in plants. Interestingly, SYMRK occurs in different forms, which define its symbiotic potential. All nodulating species, including legumes, *D. glomerata*, alder (*Alnus glutinosa*), as well as the non-nodulating species poplar (*Populus trichocarpa*) and *Tropaeolum majus*, have a long version of SYMRK with a long N-terminal extracellular region (NEC domain) and three leucine-rich repeat (LRR) motifs (Markmann et al., 2008). Non-nodulating species such as tomato (*Solanum lycopersicum*) and poppy (*Papaver rhoeas*), have a slightly shorter version with only two LRR motifs, while in the monocots, SYMRK lacks the entire NEC domain and has two LRR motifs (Markmann et al., 2008). Interestingly, only the full length SYMRK of nodulating plant species among the eurosids can fully complement nodulation in the *L. japonicus symrk* mutant, whereas the two shorter types of SYMRK complement only AM but not RNS. Surprisingly, full length SYMRK of the non-nodulating *Tropaeolum* was able to restore nodulation in *L. japonicus* (Markmann et al., 2008). This indicates that the longest version of SYMRK has gained the potential to induce bacterial accommodation in the AM-competent common ancestor of all nodulating plants, and that this ability led to the independent evolution of bacterial endosymbioses in several clades of the eurosids, whereas others (e.g., *Tropaeolum*), remained only AM-competent. Based on sequence comparison, the predisposition to bacterial symbiosis may be related to the third LRR motif in the full-length SYMRK.

### Proteins associated with symbiotic signaling components at the plasma membrane

In order to better understand the biochemical function of the receptors in symbiotic signaling, interacting protein partners have been searched for. A yeast two-hybrid screen with SYMRK yielded a MAPKK (mitogen-activated protein kinase kinase) termed SIP2, for SYMRK-interacting protein2 (Chen et al., 2012; Figure 3), which is conserved at least between *L. japonicus* and *M. truncatula* (Chen et al., 2012). MAPKKs are components of MAP kinase cascades, which are well known signal transduction pathways in plant-pathogen interactions (Tena et al., 2011). SIP2 is necessary for nodulation and may be subject to negative regulation from SYMRK in *L. japonicus* (Chen et al., 2012), indicating that fine-tuning of the MAPK cascade may be required for successful symbiosis.

Another interactor of SYMRK is the E3 ubiquitin ligase SINA4, Seven in absentia4 (Den Herder et al., 2012; Figure 3). SINA4 recruits SYMRK to small puncta at the plasma membrane that may represent microdomains dedicated to symbiotic signaling (see below). SINA4 negatively regulates SYMRK abundance and consequently modulates symbiosis signaling. In agreement with this notion, overexpression of *SINA4* leads to defects in rhizobial infection (Den Herder et al., 2012). The NFR of *M. truncatula* LYK3 interacts with PUB1 (Plant U-box E3 ubiquitin ligase1), another type of E3 ligase induced during RNS (Mbengue et al., 2010; Figure 3). LYK3, which is involved in NF selectivity (Smit et al., 2007), can phosphorylate PUB1, which in turn acts as a negative regulator of LYK3 function in infection and nodulation (Mbengue et al., 2010). Hence, PUB1 may indirectly modulate symbiosis signaling.

Interaction with several symbiosis-related receptor kinases was recently shown for remorins, a plant-specific gene family, of which at least one member, SYMREM1, is involved in nodulation (Jarsch and Ott, 2011). SYMREM1 interacts with the symbiosis receptor kinases NFP, LYK3, and DMI2 (Does not make infections2) in *M. truncatula* (Lefebvre et al., 2010), and with their respective orthologs in *L. japonicus* NFR5, NFR1, and SYMRK (Toth et al., 2012) (Figure 3). SYMREM1 is strongly and specifically upregulated in nodules and localizes to ITs, in particular at their tips where unwalled infection droplets form, and in symbiosomes (Lefebvre et al., 2010; Toth et al., 2012).

In *M. truncatula* an isoform of the isoprenoid biosynthetic enzyme HMGR (3-hydroxy-3-methylglutaryl coenzyme A reductase) was identified as an interactor of SYMRK (Kevei et al., 2007; Figure 3). Only one member of the HMGR gene family (HMGR1) interacted with SYMRK, demonstrating the specificity of the interaction. Reduction of HMGR1 activity, either by RNA interference or pharmacological inhibition of the enzyme, resulted in a strong reduction of nodulation. HMGR activity is involved in the biosynthesis of sterols, terpenoids, and in particular cytokinin, which plays an important role in RNS (Oldroyd et al., 2011). HMGR1 has two membrane-spanning domains and it localizes to small intracellular compartments of unknown identity (Kevei et al., 2007). It remains to be shown how HMGR1 interacts with SYMRK, which is localized to the plasma membrane, and what its role in nodulation is.

### Membrane microdomains as signaling platforms in symbiosis?

The plasma membrane of eukaryotes has long been thought to consist of fluid lipid bilayers in which proteins freely diffuse laterally like soluble molecules in a two-dimensional solution (Singer and Nicolson, 1972). However, biophysical as well as cell biological studies revealed that the plasma membrane is not homogeneous, but instead contains microdomains with sizes in the range of 10–100 nm in diameter that are different in their lipid and protein composition from the surrounding membrane. These microdomains are rich in sphingolipids and sterols and form platforms that can move laterally along the plasma membrane, a feature for which they were termed “lipid rafts” (Simons and Ikonen, 1997). Lipid rafts contain proteins involved in cellular signaling and membrane trafficking, whereas other general plasma membrane proteins are excluded from them. A large part of the “lipid raft” literature is based on fractionation of detergent-resistant membrane material, a technique that has been criticized for its potential to produce artifactual results (Tanner et al., 2011). We therefore focus here on proteins of which the localization has been confirmed *in vivo* with fluorescent markers or with transmission electron microscopy using immunogold labeling and we use the more generic term “microdomain” instead of “lipid rafts.”

One of the first microdomain protein markers identified in plants is remorin (Jarsch and Ott, 2011). Remorins lack a transmembrane domain or membrane anchor, hence their localization to IT and symbiosome membranes is likely to result from binding to integral membrane proteins such as LysM receptors and SYMRK (see above). The co-localization of SYMREM1 with these receptor kinases (Lefebvre et al., 2010; Toth et al., 2012) indicates that either SYMREM1 localization is a consequence of the receptors being concentrated in microdomains, or that it is involved in recruiting these receptors to microdomains, although such a mechanism has not been directly documented by fluorescently tagged proteins as in the case of SINA4 (Den Herder et al., 2012).

Recently, flotillins have been implicated in RNS (Haney and Long, 2010; Haney et al., 2011) (Figure 3). Flotillins are well conserved proteins in animals and plants (Banning et al., 2011), and like remorins, they have no membrane spanning domain, but they localize to the plasma membrane, and they are concentrated in microdomains. In *M. truncatula*, which has a flotillin gene family of seven members, *FLOT2* and *FLOT4* are required for RNS (Haney and Long, 2010). Inoculation of *M. truncatula* with rhizobia favors co-localization of FLOT4 with LYK3 in microdomains of root hairs (Haney et al., 2011). In analogy to their function in animal systems, plant flotillins may function by bringing together in microdomains components of NF signaling, thereby increasing the efficiency and perhaps the specificity of symbiosis signaling at the membrane.

Taken together, NFRs and SYMRK, together with SYMREM1, FLOT4, and SINA4, could occur primarily in membrane microdomains that serve as dedicated signaling platforms at the plasma membrane (Simon-Plas et al., 2011). The observation that down-regulation of the membrane steroid-binding protein MSBP1 in *M. truncatula* interferes with AM (Kuhn et al., 2010), indicates that regulation of sterol homeostasis may be important for AM. Given the fact that microdomains are enriched in sterols, MSBP1 could affect AM by interfering with microdomain assembly. Interestingly, signaling platforms on membrane microdomains are involved not only in symbiosis, but also in plant-pathogen interactions (Bhat et al., 2005; Keinath et al., 2010). In addition, recent evidence suggests that membrane microdomains are also involved in sugar transport (Doidy et al., 2012).

### Intracellular calcium signaling at perinuclear membranes

Besides the plasma membrane proteins involved in symbiont recognition and early signal transduction (see above), membrane proteins with essential functions in symbiosis are localized to the nuclear envelope and the ER (Figure 3). The central second messenger in symbiosis is a rhythmic calcium transient (calcium spiking) that triggers transcriptional reprogramming in host cells (Oldroyd and Downie, 2006). Calcium spiking occurs around the nucleus, suggesting that the responsible calcium channels are localized to the membrane of the nuclear envelope, and that the calcium derives from the nuclear envelope (Capoen et al., 2011). Several components of the nuclear pore complex (NPC) are required for symbiotic signaling (Parniske, 2008). Mutations in the nucleoporins (NUPs) NUP85, NUP133, and NENA lead to defective calcium spiking and aborted symbiosis (Kanamori et al., 2006; Saito et al., 2007; Groth et al., 2010). Although their role in symbiosis remains elusive, one possibility is that NUPs are involved in the translocation of membrane proteins between the inner and the outer membrane of the nuclear envelope.

The common SYM pathway also involves cation channels, DMI1 in *M. truncatula* and its homologs in *L. japonicus*, CASTOR and POLLUX, which all localize to the nuclear envelope (Riely et al., 2007; Charpentier et al., 2008; Parniske, 2008). These cation channels are thought to mediate potassium fluxes to compensate the charge imbalance resulting from calcium fluxes (Peiter et al., 2007; Charpentier et al., 2008). While the calcium channels that release the calcium are elusive, a calcium ATPase of *M. truncatula* (MCA8) has recently been described as an essential component in calcium spiking, presumably involved in reloading the calcium into the lumen of the nuclear envelope (and the ER), thereby replenishing its stores and resetting the low resting concentration of calcium in the cytoplasm and nucleoplasm (Capoen et al., 2011).

The specific calcium signatures in AM (Kosuta et al., 2008; Chabaud et al., 2011) and RNS (Oldroyd and Downie, 2006) are thought to be decoded by CCaMK (Oldroyd and Downie, 2006; Singh and Parniske, 2012). Activation of downstream transcriptional programs requires interaction with, and phosphorylation of, the CCaMK substrate CYCLOPS (Yano et al., 2008; Horvath et al., 2011). The orthologue of *CYCLOPS*, *IPD3* (Interacting Protein of DMI3), is required for symbiosis in *M. truncatula*, rice and pea (*Pisum sativum*), respectively (Messinese et al., 2007; Chen et al., 2008; Horvath et al., 2011; Ovchinnikova et al., 2011).

### Evolution of symbiotic signaling

Based on the fossil record and on the widespread occurrence of AM among the majority of vascular plants, the origin of AM is likely to have predated the radiation of land plants (Kistner and Parniske, 2002). It is conceivable that AM may even have been a precondition for successful colonization of land (Brundrett, 2002), although AM may not have been the earliest mycorrhizal association of land plants (Bidartondo et al., 2011). The finding that the common *SYM* genes are functionally conserved among mono- and dicotyledonous plant species (Chen et al., 2007, 2008; Gutjahr et al., 2008, 2012), and that they occur in lower plants such as liverworts, hornworts, mosses, and lycophytes has proven their ancient origin (Wang et al., 2010a). Interestingly, non-mycorrhizal angiosperms such as *Arabidopsis* have lost most common *SYM* genes, whereas the moss *Physcomitrella patens* has retained homologs of all *SYM* genes analyzed (Wang et al., 2010a), despite its apparent inability to undergo endosymbiosis (Wang and Qiu, 2006; Ligrone et al., 2012). It remains to be seen whether the common SYM genes of mosses play a role in other fungal associations, or whether the SYM pathway may serve other functions in the life of mosses.

Based on the fact that a number of genes are commonly required for both, AM and RNS, and that RNS ooccurs only in few taxa of the angiosperms, it was concluded that RNS evolved less than 100 Ma ago in an angiosperm predecessor that was already competent to engage in AM (Kistner and Parniske, 2002). Considering the different nodulation types, it is interesting to note that the common SYM genes are conserved also in species that form actinorhizal symbiosis (e.g., *A. glutinosa*, *C. glauca*) (Hocher et al., 2011), supporting the view that actinorrhizal symbiosis may have evolved independently from RNS in legumes, but from a common ancestor that became predisposed for bacterial symbiosis (Soltis et al., 1995; Pawlowski and Demchenko, 2012), perhaps by the modification of the LRR domain of SYMRK (Markmann et al., 2008) (see above).

The similarities between NFs and MFs (Maillet et al., 2011), and their receptors (Op Den Camp et al., 2011), also argue for a common evolutionary root of AM and RNS. In addition, the close homology of NFRs with the chitin receptor CERK1 indicates that the recognition of symbionts and pathogens derive from a common ancestral perception mechanism (Zhang et al., 2009). Since chitin, peptidoglycans and NF/MF share a common basic structure, the *N*-acetylglucosamine backbone, and since they are all perceived by LysM receptors, it is conceivable that recognition of symbiotic signals has evolved from a recognition mechanisms for an unspecific microbial signal such as chitin. The diversification of symbiotic signaling may then have been fostered by coevolution of NFRs with NFs during the evolution of RNS (Aguilar et al., 2004; Martinez-Romero, 2009). Interestingly, NFR1 and CERK1 are still so close that a few amino acid substitutions in the kinase domain of CERK1 are sufficient to confer to it the ability to induce nodules, when fused to the extracellular NF-binding domain of NFR1 (Nakagawa et al., 2011).

### How symbionts and pathogens influence their perception in plants

An open question is still why infection by AM fungi does not elicit a defense response in roots. Symbiotic plants retain, besides their NFRs and MFRs, potent receptors for microbial cell wall constituents such as chitin and peptidoglycan oligomers, which can trigger defense responses (Shimizu et al., 2010; Willmann et al., 2011). Hence, given the fact that AM fungal cell walls consist mainly of chitin, the perception of chitin fragments by plants could be expected to trigger a defense response that could block symbiosis. Indeed, some defense markers show a small transient induction at early stages of AM (García-Garrido and Ocampo, 2002), indicating that general microbe-associated molecular patterns (MAMPs) from AM fungi are perceived and elicit a transient defense response, which later is suppressed. Suppression may result from symbiotic signaling downstream of NFRs and MFRs or from manipulation by the AM fungus.

In order to escape a defense response, many microbes, beneficial and pathogenic, have evolved tools to interfere with their recognition either by hiding or by interfering with the deployment of a defense response (Zamioudis and Pieterse, 2012). The fungal pathogen *Cladosporium fulvum* has found a particularly elegant way to use the chitin-binding LysM motif to avoid its recognition: it secretes large amounts of a LysM-containing protein (Ecp6) that binds to soluble chitin fragments, thereby sequestering them from detection by the chitin receptors of the plant (Bolton et al., 2008; De Jonge et al., 2010). Hence Ecp6 is an effector protein that prevents detection of the pathogen by the host, and therefore contributes to virulence of the pathogen. Recently, an effector of an AM fungus has been described that is taken up by the host and functions through modification of defense-related gene expression in the nucleus (Kloppholz et al., 2011). It remains to be seen whether AM fungi have also tools to directly interfere with the perception of MAMPs such as chitin. Bacterial pathogens produce their own effectors to interfere with LysM receptor function, thereby preventing their detection (Gimenez-Ibanez et al., 2009; Zeng et al., 2012). Bacterial effectors are in many cases delivered directly into the cytoplasm of the host by the type-three secretion system that also exists in rhizobia (Kambara et al., 2009). Interestingly, rhizobia contain homologs of pathogen effectors that influence infectivity and host range in RNS (Lewis et al., 2011; Soto et al., 2011).

## Infection and intracellular accommodation

### Initial accommodation: infection thread and prepenetration apparatus

Intracellular accommodation of the microbial partner is the central unifying aspect of endosymbioses. In order to keep the invaded host cells intact, the microbial endosymbiont has to remain separated from the host cytoplasm by a host-derived membrane, which also has the role to control the environment of the microbe and to retrieve nutrients from it. Thus, endosymbioses require reorganization of the entire cell, in particular of the membrane system.

In order for rhizobia to invade root hair cells, the cell wall has to become locally softened and permeable. This implies a reduction in turgor pressure to avoid the plasma membrane of the host cell to rupture at the entry point. In addition, the invagination of the plasma membrane is likely to require a lowering of the turgor pressure, because the rhizobia cannot exert any inward force to promote invagination. On their way through the root hair cell, rhizobia are guided through the IT, a tubular hollow structure in which the bacteria remain confined and start to multiply. Toward the center of the root and below infected root hairs, files of cortical cells prepare for bacterial infection, before the rhizobia reach them, implying long distance transmission of a symbiotic signal (Oldroyd and Downie, 2008). Preparation of cortical cells involves migration of the nuclei to the cell center and formation of a cytoplasmic bridge through the central vacuole that traces the route for the formation of the IT, as in root hairs. This structure composed of cytoplasm and endomembranes has been termed the PIT (Van Brussel et al., 1992). On its centripetal path, the nucleus heads the PIT machinery which consists of large amounts of cytoplasm with ER thought to produce the elements of the IT (Fournier et al., 2008). Elements of the microtubular cytoskeleton are involved in the formation of the IT as well (Timmers et al., 1998). The absence of bacteria from the growing tip of the IT suggests that it is formed by the host without a direct contribution of rhizobia, although continuous signaling from the bacteria (e.g., trough NF) may influence IT development (Timmers et al., 1998; Fournier et al., 2008).

When epidermal cells are in contact with AM fungi, a similar process is triggered which consists of nuclear migration toward the contact point and assembly of an infection structure referred to as PPA. The PPA consists of dense cytoplasm with large amounts of ER cisternae, Golgi stacks, trans-Golgi network, and multivesicular bodies (Genre et al., 2005, 2008). These features of the PPA signify a strong biosynthetic activity, possibly associated with the invagination of the plasma membrane, in which the fungus inserts upon penetration of the cell wall. In *dmi2* and *dmi3* mutants, the nucleus of epidermal cells travels toward the fungal hyphopodium, but PPA formation does not occur (Genre et al., 2005), indicating that it is after nuclear migration and before PPA assembly that symbiotic signaling occurs.

### Generation of symbiotic membrane systems

The generation of the host-derived membranes associated with PIT, IT, and PPA requires *de novo* synthesis of new membrane material and of membrane proteins with specific symbiosis-related functions in signaling and transport. Intense vesicular trafficking has indeed been observed at the growing tip of ITs (Robertson and Lyttleton, 1982). Likewise, infecting hyphae of AM fungi are surrounded by dense cytoplasm with ER, numerous Golgi stacks, vesicles, and other markers of exocytotic activity (Genre et al., 2008, 2012). Finally, the formation of the arbuscules in AM and the multiplication of the bacteroids in RNS, respectively, is associated with the massive expansion of the surfaces of the PAM and of the collective symbiosome membranes (Box 1). These observations demonstrate the need for intense membrane biosynthesis and trafficking during infection and endosymbiont accommodation.

Box 1 **What is the Identity of the Periarbuscular and the Symbiosome Membranes?**

At the first intracellular stages of AM and RNS, the microbes are surrounded by the invaginated plasma membrane, and in the case of AM and some legume species that host the bacteria in fixation threads (Naisbitt et al., 1992), the PAM and the peribacteroid membrane remain continuous with the plasma membrane. The symbiosomes of most legumes, however, are isolated entities like organelles in the cytoplasm, and could therefore be compared topologically with vesicles or little vacuoles, rather than with the plasma membrane. Indeed, symbiosomes exhibit several common features with prevacuolar compartments that could turn into, or fuse with, lytic vacuoles to digest their content (Mellor, 1989). Interestingly, the analysis of membrane markers revealed an intermediate identity of the symbiosome membrane. The specific localization of the *M. truncatula* syntaxin MtSYP132 to symbiosome membranes signifies plasma membrane identity, indicating that the bacteroids reside in an “intracellular apoplastic domain” (Catalano et al., 2004, 2007; Limpens et al., 2009). Thereafter, the symbiosome membrane also carries Rab7, a marker for late endosomal/early vacuolar identity. This indicates that the symbiosome membrane goes through a phase of chimeric identity between plasma membrane and vacuolar identity (Limpens et al., 2009). At the onset of senescence, the appearance of the SNARE markers SYP22 and VTI11 signifies the identity of a lytic vacuole, in which the bacteroids are digested (Limpens et al., 2009). It is concluded that active symbiosomes are locked in a state of prevacuolar identity with contributions of plasmalemma identity. Considering the numerous symbiosis-specific features of symbiosome membrane and PAM, e.g., symbiosis-specific nutrient transporters (see main text), they may have a third, new identity which partially overlaps with plasma membrane and tonoplast identity. In addition, the PAM is subdivided into an arbuscule trunk region and the fine branches (Figure 2), which are characterized by different marker proteins (Pumplin and Harrison, 2009).

Membrane trafficking proceeds through vesicles that are fused with target membranes by a highly conserved protein machinery (Pratelli et al., 2004; Jahn and Scheller, 2006). Central players in vesicular trafficking are the SNAREs (soluble *N*-ethylmaleimide-sensitive factor attachment protein receptors), of which there are two main types: R-SNAREs (also referred to as VAMP for vesicle-associated membrane proteins) on vesicle membranes and Q-SNAREs (some called syntaxins) on target membranes such as plasma membrane or tonoplast.

### Vesicle trafficking to host-derived perimicrobial membranes

Intense cellular trafficking occurs in both mutualistic and pathogenic plant-microbe interactions (Wang and Dong, 2011; Yun and Kwon, 2012). It contributes to the local supply of new membrane material or to the delivery of cargo material (proteins or secondary metabolites) to the site of the interaction. A genetic screen in *Arabidopsis* identified PEN1/SYP121 (PENETRATION1/Syntaxin of plants121), a syntaxin with a specific role in plant immunity (Collins et al., 2003). PEN1 forms a SNARE complex with SNAP33 (Soluble *N*-ethylmaleimide-sensitive factor Adaptor Protein 33), VAMP721 and/or VAMP722, thereby providing an exocytotic delivery system for antifungal substances that contribute to full immunity in non-host resistance (Kwon et al., 2008). A related syntaxin of *Nicotiana benthamiana* (NbSYP132) plays a role in resistance against a bacterial pathogen, presumably by transporting antimicrobial proteins toward the site of bacterial infection (Kalde et al., 2007).

The symbiosome membranes of *M. truncatula* contain a syntaxin that is closely related to the aforementioned NbSYP132, namely MtSYP132 (Catalano et al., 2007). MtSYP132 may be involved in vesicle trafficking toward symbiosomes, however, the fact that it persists on the symbiosome membrane throughout its active period until senescence (Limpens et al., 2009) indicates that its function may reach beyond the generation of the symbiosome membrane, perhaps in the regulation of ion channels as it was shown for SYR1 (Syntaxin-related protein1) of *Nicotiana tabacum* (Leyman et al., 1999).

In *M. truncatula*, two vacuolar components of the quarternary SNARE complex, VAMP721d and VAMP721e, which are closely related to the PEN1 interactor VAMP721 of *Arabidopsis* (see above), play an essential role in intracellular accommodation of bacteroids and arbuscules (Ivanov et al., 2012). However, whether they interact with SYP132 on the symbiosome membrane, and what the cargo of the concerned vesicles might be, remains to be established. Taken together, these results show that in symbiosis as well as in pathogenesis of plants, a closely related machinery acts to either support intracellular accommodation of mutualistic microbes, or to fend off pathogens, respectively (Wang and Dong, 2011).

Recent evidence suggests that not all symbiosis-related factors delivered to the symbiotic interface through secretion rely on a symbiosis-specific trafficking pathway. Targeting of P transporters to the PAM may be independent of specific determinants of subcellular localization, and rather results from a general reorientation of protein trafficking from the plasma membrane toward the PAM (Pumplin et al., 2012). According to this scenario, localization to the PAM does not require specific targeting signals, but merely depends on the timing of gene expression.

### Secretion toward developing bacteroids

Further evidence for a role of protein trafficking and secretion during RNS comes from the finding that the development of functional nodules requires the signal peptidase complex, SPC (Wang et al., 2010b). Secreted or integral membrane proteins have an N-terminal signal peptide that is recognized by a signal peptide recognition particle early during translation. The nascent protein together with the ribosome is then attached to the ER, so that the protein becomes inserted into the ER membrane or transported through it. Concomitantly, the signal peptide is removed by a signal peptidase, an essential step for further processing of the protein. The mutant *defective in nitrogen fixation1* (*dnf1*) carries a mutation in the subunit SPC22 of the SPC. Although it is not the catalytic subunit, its homolog in yeast is essential for signal peptidase activity and cell growth (Fang et al., 1997). Surprisingly, *dnf1* has no developmental phenotype (Starker et al., 2006), suggesting that the function of the SPC22 subunit in *M. truncatula* is symbiosis-specific. *Dnf1* mutants accumulate nodule-specific cysteine-rich (NCR) peptides in the ER, instead of secreting them into symbiosomes, where they cause the terminal differentiation of bacteroids, a prerequisite for determinate nodule development (Van De Velde et al., 2010).

Bacteroids can differentiate in two ways which differ in their degree of determinacy. In *L. japonicus*, the bacteroids retain their morphology and reproductive capacity, i.e., they remain indeterminate, whereas in *M. truncatula*, they terminally differentiate, involving a large size increase and the inability to divide. The fate of bacteroids is thought to depend on the plant, as some rhizobia can adopt both fates in function of their host (Mergaert et al., 2006). Indeed, expression of *M. truncatula* NCR peptides in *L. japonicus* causes rhizobia to terminally differentiate (Van De Velde et al., 2010). NCR247 peptide can trigger terminal differentiation of *Sinorhizobium meliloti* also *in vitro* (Van De Velde et al., 2010). Interestingly, high concentrations of NCR247 peptide interfere with bacterial membrane integrity, thereby exerting antimicrobial activity. This effect is particularly pronounced toward *bacA* mutants of *S. meliloti*, revealing a protective effect of BacA against NCR peptides (Haag et al., 2011). As BacA is predicted to encode a cytoplasmic subunit of an ABC transporter, this protein could be involved in either the uptake or efflux of NCR peptides in order to prevent plasma membrane damage (Haag et al., 2011). *BacA* mutants are protected in the *dnf1* mutant because NCRs are retained in the ER. These results show that RNS does not represent perfect harmony but rather a balance between cooperation and control.

### Roles of organelles in symbiosis

Cells with arbuscules and symbiosomes generally contain large amounts of organelles, indicative of intense metabolic activity (Figures 1 and 2). The plastids in mycorrhizal cells are of particular interest because they are closely associated with the arbuscules, and they considerably change their shape to a network-like system, referred to as stromules (Lohse et al., 2005, 2006; Strack and Fester, 2006). The plastids of mycorrhizal roots are active in carotenoid and apocarotenoid metabolism, which may be significant for symbiosis due to their role in the biosynthesis of the hormones gibberellin, ABA and strigolactone (Walter et al., 2010). Furthermore, plastids serve as factories for fatty acid biosynthesis, which is a prerequisite for the expansion of the membrane systems in symbiotic cells.

During AM and RNS the large central vacuole of colonized cells fragments to yield room for the accommodation of the symbiont (Cox and Sanders, 1974; Bonfante-Fasolo, 1984; Van Brussel et al., 1992; Hause and Fester, 2005). The close association of the symbiosome membrane with the tonoplast in mycorrhizal cells (Figure 1) may indicate a role of vacuolar membranes or vacuolar constituents in symbiosis.

### A new cellular compartment involved in symbiosis?

In a genetic screen for mutants affected in intracellular accommodation of AM fungi in *P. hybrida*, the mutant *penetration and arbuscule morphogenesis1* (*pam1*), was isolated (Sekhara Reddy et al., 2007). *PAM1* encodes a novel plant-specific protein with an N-terminal major sperm protein (MSP) domain that is also found in the VAMP-associated proteins (VAPs) which are involved in vesicle trafficking (Lev et al., 2008; Feddermann et al., 2010). The C-terminus consists of 11 ankyrin repeats (Feddermann and Reinhardt, 2011), which are involved in protein–protein interactions in eukaryotes (Bennett and Baines, 2001; Mosavi et al., 2004). Due to this domain structure, the protein is referred to as VAPYRIN. VAPYRIN homologs were found in almost all plant species, including the non-symbiotic moss *P. patens*, but with the notable exception of *A. thaliana*. Functional conservation of VAPYRIN was shown in *M. truncatula*, where *vapyrin* mutants are defective in both AM and in RNS, indicating that intracellular accommodation, like the common SYM pathway, is shared between bacterial and fungal endosymbioses (Pumplin et al., 2010; Murray et al., 2011). The fact that calcium spiking is normal in *vapyrin* mutants shows that VAPYRIN acts downstream of the calcium signal and perhaps of the entire common SYM signaling pathway (Murray et al., 2011).

Petunia VAPYRIN localizes to the nucleus and the cytoplasm, with a conspicuous accumulation to mobile spherical structures that are associated with the tonoplast, and therefore termed tonospheres (Feddermann et al., 2010). In AM of petunia, tonospheres associate with fungal hyphae (Feddermann et al., 2010). In *M. truncatula*, mobile puncta with VAPYRIN-GFP protein that probably correspond to tonospheres, accumulate exclusively in colonized cells (Pumplin et al., 2010). VAPYRIN does not contain a signal peptide, nor any predicted transmembrane domain, indicating that the association with membranes is likely to result from protein–protein interaction with resident membrane proteins (Feddermann and Reinhardt, 2011).

## Membrane transporters in symbiosis

The “raison d'être” of endosymbioses is the exchange of nutrients representing a mutual benefit to both symbiotic partners (Box 2). In RNS this involves primarily the transfer of N in the form of ammonium from bacteroids to the plant, and the reverse transfer of dicarboxylic acids such as malate, fumarate, or succinate to the bacteroids (Prell and Poole, 2006). In the case of AM, there is a range of nutrients that AM fungi can deliver to plants, with the most prominent examples of P, N, and S (Allen and Shachar-Hill, 2009; Smith and Smith, 2011). However, AM fungi can also acquire water and micronutrients from the soil and deliver them to the plant host in exchange for fixed C (Clark and Zeto, 2000).

Box 2 **Are Nutritional Fluxes between Plants and AM Fungi Interrelated?**

The ancient origin and the wide distribution of AM raises the question how mutualism has been stabilized over evolutionary time, but also during ontogenetic development. Mutualism requires a high degree of coordination and synchronization between the partners, and is prone to exploitation by one or the other, leading to a parasitic or pathogenic interaction. Indeed, heterotrophic (achlorophyllous) plants have in multiple independent cases turned into parasites of AM fungi (Merckx et al., 2012). In a less extreme converse scenario, an AM interaction can result in a negative growth effect on the plant reflecting a parasitic relationship where the costs of the interaction exceed the benefit for the plant (Li et al., 2008). However, in most cases, AM are mutualistic, and exploitation is the exception.

How is mutualism stabilized in AM? One possibility is that the partners prevent exploitation by imposing sanctions on their partner in case of reduced symbiotic service. There are indeed indications for such a scenario: for example, the flux of P_i_ toward the plant influences to which extent the fungus is allowed to proliferate in the root. If P_i_ delivery is blocked by a mutation in the PT of the plant, fungal colonization is reduced, and intracellular fungal structures are subject to premature senescence (Maeda et al., 2006; Javot et al., 2007). On the other end of the scale, high levels of P_i_ also repress AM fungal colonization (Breuillin et al., 2010). In both cases, the phenotype is different than in common *SYM* mutants, indicating that symbiosis is blocked at a rather late stage. However, high P_i_ supply is also known to impact on early signaling through the inhibition of strigolactone biosynthesis (Balzergue et al., 2011).

Direct insight into sanctions come from measurements of nutrient flux in monoxenic root cultures as a function of nutrient supply and environmental conditions. For example, plants can reduce C allocation to AM fungi, when they are supplied with optimal P_i_ levels through fertilization (Olsson et al., 2010). Conversely, when AM are supplied with limited C levels, P_i_ accumulates in the AM fungus instead of being transferred to the plant (Hammer et al., 2011). However, the question arises, whether in a more natural setting, when plants and AM fungi occur simultaneously in combinations with several potential partners, plants and AM fungi can selectively identify and reward better mutualists. This question was tested in an elegant approach, where isotope incorporation into newly synthesized fungal RNA allowed the separation of material from different AM fungi hosted by the same plant, allowing to estimate their relative consumption of sugar from the plant (Kiers et al., 2011). These experiments directly showed that plants colonized by two AM fungi preferentially reward the fungus that provides more P_i_ (Kiers et al., 2011). Conversely, AM fungi allocate P_i_ and N preferentially to roots that supply them with more C (Fellbaum et al., 2011; Kiers et al., 2011). These results document that AM involve a bidirectional rewarding system which can be considered a “biological market” and which is believed to help maintain mutualism within individual AM interactions and over evolutionary times.

### Water relations and aquaporins

AM fungi can increase the drought resistance of plants in several ways. Firstly, some AM fungi can considerably promote water uptake of mycorrhizal plants (Marulanda et al., 2003), and they can prevent leaf dehydration during drought and salt stress (Aroca et al., 2007). Furthermore, mycorrhizal plants have a lower and more stable root hydraulic conductance than non-mycorrhizal plants, leading to increased water use efficiency (WUE) that is higher amounts of photosynthate generated per volume of acquired water (Augé, 2001). Improved water relations may result from a generally improved nutritional status, but direct effects of AM fungi on water uptake and transport have also been reported (Marulanda et al., 2003; Egerton-Warburton et al., 2007).

In principle, water flux across membranes proceeds passively through osmosis along proteinaceous pores that facilitate water diffusion through the membrane (Zeuthen, 2010). Aquaporins facilitate water transfer through membranes along an osmotic gradient, but they cannot actively pump water against a water potential gradient. In plants, aquaporins occur as exceptionally large and diverse gene families, suggesting that they play important roles in various processes of plant life (Maurel et al., 2009; Anderberg et al., 2012). Aquaporins of higher plants are classified into five groups, according to their subcellular localization, expression pattern, and protein structure: plasma membrane intrinsic proteins (PIPs), tonoplast intrinsic proteins (TIPs), nodulin26-like intrinsic proteins (NIPs), small and basic intrinsic proteins (SIPs), and X intrinsic proteins (XIPs) (Danielson and Johanson, 2010).

Besides their function as water channels, aquaporins have been shown to facilitate the diffusion across membranes of low molecular weight neutral solutes such as glycerol, ammonia, and carbon dioxide (Dean et al., 1999; Uehlein et al., 2008; Hwang et al., 2010). Consistent with a role in endosymbiosis, several members of the PIP-, TIP-, and NIP-subfamilies are induced in both AM and RNS in rice, *M. truncatula*, *L. japonicus*, and petunia (Güimil et al., 2005; Hohnjec et al., 2005; Guether et al., 2009a; Breuillin et al., 2010). In particular the NIP NOD26, which can account for 10% of the total symbiosome membrane protein (Rivers et al., 1997; Catalano et al., 2004) is of considerable interest. With its ammonia transport activity, NOD26 would be well suited to allow for N transfer along the source to sink gradient between bacteroids and plant. The fact that *NOD26* is also induced in AM (Güimil et al., 2005; Hohnjec et al., 2005; Guether et al., 2009a; Breuillin et al., 2010) is in line with the finding that AM fungi, like rhizobia, release N to the plant host in the form of ammonia (Govindarajulu et al., 2005). However, it should be noted that in the acidic environment of the symbiotic interface around arbuscules and bacteroids, ammonia is almost completely protonated to the charged form ammonium (NH^+^_4_, pKb = 9.25), for which permeability in NOD26 has not been shown. Hence N uptake from the symbiotic interface into the host cytoplasm is more likely to be mediated by ammonium transporters (see below) than by NOD26.

In addition to their transport activity, aquaporins can mediate close interactions of juxtaposed membranes for example in the lens of mammals (Engel et al., 2008). Vacuolar subcompartments with multiple membrane layers and high contents of γ- and δ-TIP were observed in *Arabidopsis* cotyledons (Saito et al., 2002). These structures are highly mobile and move along the inner surface of the tonoplast to which they remain attached. Similar mobile structures were identified in mycorrhizal roots, where they contain the VAPYRIN protein (see above). Despite a number of reports about the involvement of aquaporins in AM and RNS, their exact biochemical function in symbiosis, as in many processes of plant development, remains to be established (Hill et al., 2004).

### H^+^-ATPases

In contrast to the aquaporins, mineral nutrient transporters require an active transport mechanism, since they often act against a concentration gradient. Most nutrient transporters in the plasma membrane use the energy of the proton electrochemical gradient generated by H^+^-ATPases. In the direct (non-symbiotic) nutrient uptake pathway, plants acquire nutrients from the soil, whereas in the indirect mycorrhizal pathway, nutrients are taken up from the periarbuscular space over the PAM. In both cases, transport is energized by proton gradients. H^+^-ATPases are induced in AM (Gianinazzi-Pearson et al., 2000; Krajinski et al., 2002) and are thought to contribute to the uptake of inorganic phosphate (P_i_) and other nutrients from the symbiotic interface by proton symport (Karandashov and Bucher, 2005). Indeed, the periarbuscular space is acidified (Guttenberger, 2000), an observation which is compatible with the localization of an ATPase activity at the PAM (Marx et al., 1982). Hence, to energize nutrient uptake from the symbiotic interface, plants generate a proton gradient (Ferrol et al., 2002) to which the mycorrhizal fungus may also contribute (Requena et al., 2003; Breuninger and Requena, 2004; Balestrini and Lanfranco, 2007; Ramos et al., 2009). An activity analogous to the H^+^-ATPase in the PAM was identified at the symbiosome membrane, which provides both the plant and the bacteroids with an electrochemical gradient for nutrient uptake from the peribacteroid space (Fedorova et al., 1999; Saalbach et al., 2002; Catalano et al., 2004).

### Phosphate transport

The most thoroughly studied nutrient transport pathway in AM is the transport of P_i_ (Karandashov and Bucher, 2005) which is taken up from the soil by fungal P_i_ transporters (PTs) (Harrison and Van Buuren, 1995; Maldonado-Mendoza et al., 2001; Requena et al., 2003; Benedetto et al., 2005). Surprisingly, a PT of *Glomus mossae* (*GmosPT*) is expressed at similar levels in the extraradical and intraradical mycelium (Benedetto et al., 2005). Hence, the AM fungus could potentially control P_i_ flux toward the plant by partial re-uptake of P_i_ from the root (Benedetto et al., 2005; Balestrini et al., 2007). AM fungi store P_i_ as polyphosphate in tubular vacuoles (Uetake et al., 2002; Kuga et al., 2008; Olsson et al., 2011). Polyphosphate is a linear P_i_ polymer that can comprise thousands of P_i_ residues. Polyphosphate as vacuolar storage form helps to keep P_i_ levels in a physiological range in the fungal cytoplasm, and prevents osmotic effects. Furthermore, the low cytoplasmic concentration of free P_i_ favors further P_i_ uptake from the soil. Polyphosphate is translocated in mobile vacuoles from the extraradical mycelium to the mycorrhizal roots (Maldonado-Mendoza et al., 2001; Hijikata et al., 2010), and released as free P_i_ into the periarbuscular space, where it is taken up by plant PTs.

The best-characterized symbiotic PT of plants is the *M. truncatula* AM-specific low-affinity transporter MtPT4, which is localized exclusively to the PAM (Harrison et al., 2002). MtPT4 activity is required not only for improved shoot P_i_ status, but also for sustained AM colonization of the root system (Javot et al., 2007). In *mtpt4* mutants, arbuscules accumulate polyphosphate, indicative of an impairment of P_i_ transfer, and they senesce prematurely. Thus plants can sense the quality of symbiotic service and sustain or terminate symbiosis, depending on the resulting benefit (Javot et al., 2007). *Solanaceae* such as tomato and petunia have three AM-responsive *PT* genes (*PT3*-*PT5*) among which *PT4* is the only AM-specific one (Wegmüller et al., 2008; Nagy et al., 2009). This redundancy complicates functional analysis compared to *M. truncatula*. In mycorrhizal tomato roots, high levels of *LePT3* and *LePT4* were detected in arbuscule-containing cells (Balestrini et al., 2007). In potato, the related *StPT3* gene is active in cells with arbuscules as with hyphal coils (Rausch et al., 2001; Karandashov et al., 2004), consistent with active P_i_ uptake in colonized cells of both *Arum*- and *Paris*-type AM. Expression of the AM-specific low-affinity rice PT *OsPT11* is correlated with the degree of *G. intraradices* colonization, as MtPT4 (Harrison et al., 2002; Paszkowski et al., 2002; Kobae and Hata, 2010). OsPT11, which was studied in both *Arum*-type and *Paris*-type mycorrhiza, localizes exclusively to the membrane around branched hyphae, but not at the plasma membrane neither around hyphal coils or hyphal trunks, a pattern similar to MtPT4 (Harrison et al., 2002; Pumplin and Harrison, 2009; Kobae and Hata, 2010). Hence, the expression pattern and the subcellular localization of AM-specific PTs in mono- and dicotyledonous plants reveals a conserved P_i_ uptake pathway in colonized cells of *Arum*- and *Paris*-type AM.

Interestingly, symbiosis-related PTs of monocots [e.g., OsPT11 of rice and ZmPT6 of maize (*Zea mais*)] and dicots (e.g., MtPT4 of *M. truncatula* and LePT4 of tomato) share a common phylogenetic root with the PT families of the lower land plants *P. patens* and *Selaginella moellendorffii*, documenting their common ancestral origin relative to the more derived members of the constitutive P_i_ uptake pathway in angiosperms (Paszkowski, pers. communication). A close evolutionary relationship among the symbiosis-specific PT is also documented by the conservation of their promoter sequences relative to related PTs that are induced by AM to a lesser degree, such as the PT3 lineage of the *Solanaceae* (Rausch et al., 2001; Chen et al., 2011).

### Nitrogen transport

AM fungi, like roots, can acquire N from the soil primarily as nitrate (NO^−^_3_) or as ammonium (NH^+^_4_) (Tian et al., 2010), although organic forms may also be involved (Cappellazzo et al., 2008). Two ammonium transporters, GintAMT1 and GinAMT2, were identified in *Glomus intraradices*. The high affinity transporter GintAMT1 is substrate-inducible and is expressed preferentially in the extraradical mycelium (Lopez-Pedrosa et al., 2006; Perez-Tienda et al., 2011), whereas GintAMT2 is preferentially expressed in the intraradical mycelium and is not substrate-regulated (Perez-Tienda et al., 2011). Interestingly, GintAMT1 and GintAMT2 are both expressed in arbuscule-containing cells (Perez-Tienda et al., 2011), indicating that they may modulate the amount of delivered N by reuptake, as it has been proposed for P_i_ transport (see above).

Once in the extraradical mycelium, N is thought to be translocated in the form of arginine which carries four N atoms per molecule and therefore represents a concentrated transport form of N (Govindarajulu et al., 2005). The fate of N from the soil to the plant through the AM fungus has been well described through the analysis of the enzymatic steps of ammonium assimilation, arginin biosynthesis in the extraradical hyphae, and arginine catabolism in intraradical hyphae (Govindarajulu et al., 2005; Tian et al., 2010). N is then thought to be transferred to the periarbuscular space in an inorganic form probably as ammonium, which can be taken up by the PAM via ammonium transporters such as LjAMT2.2 in *L. japonicus* (Guether et al., 2009b), GmAMT4.1 in soybean (*Glycine max*) (Kobae et al., 2010), and their homologs in *M. truncatula* (Gomez et al., 2009; Gaude et al., 2012).

Like AM fungi, the bacteroids in nodules release fixed N in the form of ammonia which is taken up by ammonium transporters in the symbiosome membrane (Kaiser et al., 1998; Rogato et al., 2008). Whether the ammonia-permeable aquaporin NOD26 plays a prominent role in N uptake of plants, as suggested by Hwang et al. (2010) is not clear (see above). However, patch clamp experiments have revealed a channel-like activity through which ammonium from the peribacteroid space can be taken up into the cytoplasm of the host (Tyerman et al., 1995;Kaiser et al., 1998).

### Carbohydrate transport

Recent progress has significantly advanced our understanding of sugar transport within plants and in the interaction with beneficial and pathogenic microbes (Doidy et al., 2012). For nutrition of endosymbiotic microbes, two sugar transport steps are required. First, symbiotic tissues such as nodules and mycorrhizal roots need to attract photosynthate in competition with other sinks, and they need to take up sugar either directly from the phloem, or from the surrounding apoplast. Secondly, symbiotic cells need to release an appropriate form of C to the microbe at the symbiotic interface. In plants, the mobile form of reduced C is primarily sucrose which is cleaved to hexoses (glucose and fructose) in sink tissues. Hence, sink tissues of plants can acquire carbohydrate either by sucrose transporters or by monosaccharide (hexose) transporters. Candidates for sink-related transporters in symbiosis are the AM-inducible hexose transporter Mtst1 in *M. truncatula* (Harrison, 1996), and the sucrose transporter *LjSUT4* induced during nodule development in *L. japonicus* (Flemetakis et al., 2003).

It has long been an open question how hexoses may be released from cells in general, and from AM colonized cortex cells in particular. Only recently, a family of plant hexose transporters has been identified (SWEET) that can serve for hexose export from cells (Chen et al., 2010). SWEETs are uniporters that can transfer hexoses in both directions, depending on the sugar gradient over the plasma membrane. In animals, SWEETs release hexoses to extracellular compartments such as the blood (Chen et al., 2010). *A. thaliana* has 17 SWEETs, suggesting that they play diverse roles in plant life. SWEETs can be exploited by pathogens for their own nutrition (Chen et al., 2010). Interestingly, the nodule-specific MtN3 is a member of the *M. truncatula* SWEET family, and may therefore be involved in the nutrition of the bacteroids in nodules. Whether members of the SWEET family indeed play a role in AM or RNS remains to be shown.

Hexoses are the likely transfer form to supply the heterotrophic AM fungus with fixed C (Pfeffer et al., 1999). AM fungi have a hexose transporter, MST2, that can take up glucose, galactose, mannose and the oxidized sugars glucuronic and galacturonic acid (Helber et al., 2011). MST2 is required for fungal proliferation in roots, indicating that it is involved in nutrition of the fungus during symbiosis (Helber et al., 2011).

The C source provided to bacteroids in nodules consists of dicarboxylic acids (Long, 1989). Indeed, *S. meliloti* possesses a dicarboxylate transporter, DctA, which is suggested to transport several compounds, mainly malate and fumarate (Yurgel and Kahn, 2005). DctA is required for RNS, in particular for the energy-demanding N fixation by bacteroids, since *dctA* mutants are impaired in N fixation.

### Other transporters with potential roles in symbiosis

Some proteins are required for the establishment of a functional endosymbiosis, but their cellular and biochemical function remains elusive. For instance the *L. japonicus* mutant *sen1* can form nodules when colonized with *Mesorhizobium loti*, but the nodules remain pale and small, and N fixation is abolished (Hakoyama et al., 2012). SEN1, which is expressed specifically in the infected cells of nodules, is homologous to vacuolar cation transporters for iron and manganese. It is conceivable that a depletion in iron or manganese may hamper N fixation in bacteroids since iron is required for the nitrogenase complex, apart from general bacteroid metabolism (Hakoyama et al., 2012).

In AM, a likely candidate for a sulfate transporter was recently identified in *M. truncatula* (Casieri et al., 2012), however, its functional relevance in symbiosis, as well as its subcellular localization remain to be established. Two half-size ABC transporters of the same species, STUNTED ARBUSCULE (MtSTR) and MtSTR2 are essential for functional arbuscules (Zhang et al., 2010). *MtSTR* and *MtSTR2* are expressed specifically in arbuscule-containing cells, where they localize to the PAM. MtSTR and MtSTR2 were found to heterodimerize creating a full-size transporter that is localized to the PAM around young and mature arbuscules, but not around the hyphal trunk. Homologs of MtSTR and MtSTR2 were found in rice (*OsSTR1* and *OsSTR2*; Gutjahr et al., 2012), but not in the non-symbiotic model species *A. thaliana*, consistent with a specific role in symbiosis. However, their function in symbiosis remains elusive since their substrates are unknown.

## Conclusions

Membranes are a central feature of life, since they allow the interior of cells to establish controlled conditions separated from the environment to provide optimal conditions for biochemical processes. In endosymbiosis, this aspect is accentuated, since two organisms cooperate in such close proximity that not much more than a membrane, a thin cell wall, and some interstitial material separates their cytoplasms. Therefore, highly organized membrane systems are at the core of endosymbioses. They are involved at all levels from initial recognition over intracellular accommodation to the establishment of the symbiotic interface, over which nutrients are exchanged. A topic that will attract increasing interest in coming years is the compartmentalization of the plasma membrane and the peri-microbial membranes (PAM and symbiosome membrane). An emerging scenario is that plants—like animals—have membrane microdomains that serve as signaling platforms in symbiosis and in plant-pathogen interactions. These membrane microdomains contain receptors and signaling components that are subject to dynamic regulation in space and time. Also, the emerging notion that the recognition of microbial pathogens and symbionts by LysM-containing receptors may share a common origin from non-self recognition mechanisms line out exciting new avenues for future research. Comparison of the molecular basis of symbiotic signaling and development in different taxa will help elucidate the evolution of AM in the ancestors of vascular plants, and the multiple emergence of RNS in a predisposed monophyletic clade within the angiosperms.

### Conflict of interest statement

The authors declare that the research was conducted in the absence of any commercial or financial relationships that could be construed as a potential conflict of interest.

## Appendix

**Table A1:** **Genes involved in regulation of arbuscular mycorrhiza and/or root nodule symbiosis**.

**Genes**	**Organism**	**Structural characteristics**	**(putative) Localization**	**Mutants/transformed lines analyzed**	**phenotypes of the mutants/transformed lines**	**Involvement in**	**(putative) Function**	**References**
(Ph)PDR1	*P. hybrida*	ATP-binding cassette transporter subtype G (ABCG)	Plasma membrane	Transposon insertion mutant and KD RNAi lines in petunia plants; *A. thaliana* OE lines	Silenced lines: delay in AM colonization, strigolactone exudate levels affected; *A. thaliana* OE lines: higher tolerance to the synthetic strigolactone less retained into roots.	AM	Strigolactone exporter	Kretzschmar et al., 2012
(Lj)NFR1/(Mt)LYK3	*L. japonicus*/*M. truncatula*	LYK	Plasma membrane	(1,2,3) Mutants from a T-DNA insertion screen (*Ljnfr1-1/Ljsym1-1, Ljnfr1-2/Ljsym1-2*) (4) *M. truncatula* EMS-induced mutants *B56/hcl-1, W1/hcl-2, AF3/hcl-3* (5) KD RNAi transformed roots. (6) *M. truncatula* EMS-induced mutants *hcl-1, hcl-2, hcl-3, AC6/hcl-4/lyk3-4*	(1,2) Nod- phenotype, AM not affected (Ami+). (3) No root hair deformation, no root hair curling, no infection threads nor nodule primordia induced. (4) *HCL* stands for defective in root hair curling; only root hair deformation and reduced cortical cell division without meristematic cells. (5) Upon inoculation with a rhizobia strain mutated for the NF: root hair curling but decrease of IT number, ITs with a sac-like structure aborted in the root hair. (6) Concerning the fourth *lyk3* mutant *hcl-4*: normal root hair curling observed, ITs with a sac-like shape and often aborted, few nodules.	RNS	NF receptor	(1) Schauser et al., 1998; (2) Wegel et al., 1998; (3) Radutoiu et al., 2003; (4) Catoira et al., 2001; (5) (Limpens et al., 2003); (6) Smit et al., 2007
(Lj)NFR5/(Mt)NFP/(Pa)NFP	*L. japonicus*/*M. truncatula*/*P. andersonii*	LYK	Plasma membrane	(1,3,4,5) Mutant from a T-DNA insertion mutants screen (*282-894/Ljnfr5-1/Ljsym5*), Ac-TE insertion mutant (*Ljnfr5-2*), (2) EMS-induced mutant (*EMS223/Ljnfr5-3/Ljsym25*); (6,7) EMS-induced mutants (*Mtnfp-1, Mtnfp-2*), KD RNAi transformed roots; (8) KD RNAi transformed roots (*PaNFP*)	(1,2) Nod- phenotype. (1,3) Ami+ phenotypes. (2) (4, 5) Nod- phenotype and unresponsiveness to inoculation with *M. loti* or application of NF (no root hair deformation). (6,7) EMS-induced mutants: Nod- phenotype, absolutely no sign of RNS, even root hair deformation. (7) Silenced lines: ITs abortions in root hair cells, with sac-like structures. (8) Impairment of both RNS and AM.	RNS (and AM for *P. andersonii*)	NF receptor	(1) Schauser et al., 1998; (2) Szczyglowski et al., 1998 (3) Wegel et al., 1998; (4) Madsen et al., 2003; (5) Radutoiu et al., 2003; (6) Ben Amor et al., 2003; (7) Arrighi et al., 2006; (8) Op Den Camp et al., 2011
(At)CERK1/(Os)CERK1	*A. thaliana*/*O. sativa*	LYK	Plasma membrane	(1) KO transposon and T-DNA insertion mutants *Atcerk1-1* and *Atcerk1-2*; (2,6) KO T-DNA insertion mutant *Atcerk1-2*; (3,4) KO T-DNA *Atcerk1-2* and *Atcerk1-3* mutants; (5) KD RNAi OsCERK1 lines	(1) No responses to chitin, including MAPK activation, ROS generation, and gene expression; impairment of disease resistance against the incompatible fungus *Alternaria brassicicola*. (2) Impairment of chitin-responsive genes, increase of fungal but not of bacterial growth (*Erysiphe cichoracearum* and *Alternaria brassicicola*; and *Pto* DC3000 respectively). (3) Enhanced growth of the virulent bacterial strain *Pto* DC3000 and the non-pathogenic strain *Pto* DC3000 *hrcC*. (4) Impairment of FRK1 induction after chitin and PGN treatment, as well as PGN-responsive genes. (5) Impairment of specific ROS generation and of phytoalexins accumulation. (6) Impairment of ROS generation, defense-related genes.	Defense	Chitin and PGN receptor	(1) Miya et al., 2007; (2) Wan et al., 2008; (3) Gimenez-Ibanez et al., 2009; (4) Willmann et al., 2011; (5) Shimizu et al., 2010; (6) Shinya et al., 2012
(At)LYM2/(Os)CEBiP	*A. thaliana*/*O. sativa*	LYM	Plasma membrane	(1) OsCEBiP KO RNAi lines; (2) transposon insertion mutants *lym2-2*, *lym2-3*	(1) Suppression of the specific ROS generation and impairment of gene responses induced by chitin. (2) No impairment of ROS generation.	Defense	Chitin receptor	(1) Kaku et al., 2006; (2) Shinya et al., 2012
(At)LYK4	*A. thaliana*	LYK	Plasma membrane	T-DNA insertion mutant *lyk4*	Impairment of chitin-responsive genes, increase of fungal and bacterial growth (*Alternaria brassicicola* and *Pto* DC3000 respectively).	Defense	Chitin receptor	Wan et al., 2012
(At)LYM1	*A. thaliana*	LYM	Plasma membrane	(1) T-DNA insertion mutants *lym1-1*, *lym1-2*; (2) T-DNA insertion mutant *lym1-1*, transposon insertion mutant *lym1-2*	Impairment of FRK1 induction after PGN treatment, as well as PGN-responsive genes; enhanced growth of *Pto* DC3000. (2) No impairment of ROS generation (*lym1-2*).	Defense	PGN receptor	(1) Willmann et al., 2011; (2) Shinya et al., 2012
(At)LYM3	*A. thaliana*	LYM	Plasma membrane	(1,2) cs *lym3-1*, *lym3-2*	(1) Impairment of FRK1 induction after PGN treatment, as well as PGN-responsive genes; enhanced growth of *Pto* DC3000, *Pto* DC3000 hrcC- and the hypovirulent *Pto* DC3000 Δ avrPto/PtoB. (2) No impairment of ROS generation (*lym3-1*).	Defense	PGN receptor	(1) Willmann et al., 2011; (2) Shinya et al., 2012
(Lj)SYMRK/(Ms)NORK/(Mt)DMI2	*L. japonicus*/*M. sativa*/*M. truncatula*	LRR-RLK	Plasma membrane and IT	(1,2,7) *L. japonicus* EMS-induced mutant (*EMS61/Ljsym21-2*) and (1,3,4,7) mutants from a transposon/T-DNA insertion mutants screen (*282-287/Ljsym2-1, cac41.5*); (5,6) *M. sativa* MN-1008 mutant obtained from crosses between different cultivars; (8) *M. truncatula* KD RNAi transformed roots, transformed roots with the RNAi hairpin construct *ENOD12::DMI2i*, and *35S::DMI2*-transformed roots from the TR25 mutant (lowering DMI2 activity in the nodule apex)	(1) Nod- phenotype. (2) Amplified swelling and branching, but no curling of root hairs when inoculated with *M. loti*. No more induction of the leghaemoglobin gene. (3,4) Coi- and Nod- phenotypes. (5,6,7) Myc- and Nod- phenotypes. (8) RNAi transformed roots: few nodules formed, in which ITs but few symbiosomes were observed; transformed roots with the RNAi hairpin or *35S::DMI2* constructs: more nodules formed, ITs growth amplified, with enlarged and branched ITs, but no symbiosomes observed, Fix- phenotype.	Common SYM pathway	Required for accommodation in both symbioses; positioned between NF perception and calcium spiking in the common SYM pathway	(1) Szczyglowski et al., 1998 (2) Stracke et al., 2002; (3) Schauser et al., 1998; (4) Wegel et al., 1998; (5) Caetano-Anollés and Gresshoff, 1991; (6) Endre et al., 2002; (7) Kistner et al., 2005; (8) Limpens et al., 2005
(Lj)SIP2	*L. japonicus*	MAPKK	Plasma membrane-associated and cytoplasm	KD RNAi transformed roots	Strong down-regulation of three marker genes for IT and nodule primordium formation; impairment of IT and nodulation formation.	RNS	Functional MAPKK interacting with SYMRK and that could be involved in the regulation of early symbiotic signal transduction and nodule organogenesis may be due to the inhibitory effect of SYMRK on its activity	Chen et al., 2012
(Lj)SINA4	*L. japonicus*	E3 ubiquitin ligase	Cytoplasm	OE transformed roots and OE transgenic lines	OE transformed roots: reduced SYMRK protein levels upon *M. loti* inoculation; OE transgenic lines: decrease of infection events and number of ITs, increase of white nodules, thick and branched ITs in both white and pink nodules, no or few bacteroids observed.	RNS	SYMRK turnover	Den Herder et al., 2012
(Mt)PUB1	*M. truncatula*	E3 ubiquitin ligase	Plasma membrane-associated	OE and KD RNAi transformed roots in the wild-type background and in the *hcl-4 (lyk3-4)* background	OE transformed roots: delay in nodulation observed; KD transformed roots (wt background): number of nodules strongly increased only upon *S. meliloti* mutant strains (*nodL, nodFnodL*: NF modified at the non-reducing end); KD transformed roots (*hcl-4* background): impairment of nodulation and IT development observed in *hcl-4* mutant overcame.	RNS^*^	Functional E3 ubiquitin ligase that interacts with LYK3 physically and functionally by regulating negatively infection and nodulation	Mbengue et al., 2010
(Lj)SYMREM1/(Mt)SYMREM1	*L. japonicus*/*M. truncatula*	Remorin	Host-derived membrane-associated with a localization to nodular ITs, more strongly at the tip where unwalled infection droplets form, and symbiosomes	(1) KD RNAi transformed roots, stable RNAi lines, *Tnt1*-transposon insertion mutants; (2) OE transformed roots	(1) RNAi transformed roots: no or few nodulation, often with small and white nodules, multiplication of IT formation, IT often aborted, highly branched or with a sac-like structure. Stable RNAi lines: enlargement of ITs and absence of symbiosome, suggesting a delay in rhizobia release into host cells; KO line: morphological change of nodule shape, enlarged and highly branched ITs, almost no symbiosomes formed. (2) Increase of mature nodules number but not in IT number.	RNS	Role in RNS accommodation and hypothesized to supervise localization, sorting and regulation of LjNFR1/MtLYK3, LjNFR5/MtNFP and LjSYMRK/MtDMI2 during RN symbiosis in plasma membrane sub-domains.	(1) Lefebvre et al., 2010; (2) Toth et al., 2012
(Mt)HMGR1	*M. truncatula*	3-Hydroxy-3-Methylglutaryl Coenzyme A Reductase	Vesicle-like structures	Pharmacological inhibition and KD RNAi transformed roots	Pharmacological inhibition: decrease of nodule number; KD transformed roots: nodulation strongly decreased, with ITs and nodules development arrested at an early step, Fix- phenotype.	RNS^*^	HMGR1 activity is required for nodule development; four hypotheses were mentioned concerning the recruitment of HMGR1 by NORK and the link with the mevalonic acid (MVA) pathway	Kevei et al., 2007
(Mt)FLOT2	*M. truncatula*	Flotillin-like	Plasma membrane-associated microdomains	KD RNAi and amiRNA transformed roots	Few infection events, normal IT development but great part of small and white nodules; also decrease in primary root length as well as increase in lateral root length.	RNS	Required for IT initiation	Haney and Long, 2010
(Mt)FLOT4	*M. truncatula*	Flotillin-like	Plasma membrane-associated microdomains	KD RNAi and amiRNA transformed roots	Few infection events, ITs largely aborted in root hair, great part of small and white nodules; also increase in the number of secondary lateral roots.	RNS	Required for IT initiation and development	Haney and Long, 2010
(Mt)MSBP1	*M. truncatula*	Membrane-bound steroid-binding protein	Nuclear membranes and surrounding ER	Transformed roots with KD RNAi lines	KD lines: frequency of mycorrhizal colonization unchanged but infection sites often aborted. In case of successful infection sites, septated hyphae and collapsed arbuscules observed.	AM	Sterol homeostasis	Kuhn et al., 2010
(Lj)CASTOR and (Lj)POLLUX/(Mt)DMI1	*L. japonicus*/*M. truncatula*	Ion channel	Nuclear membranes, (5) preferentially the inner nuclear membrane	(3,4,5,6) EMS-induced mutant (*EMS1749/Ljsym4-2*); (2,3,4,5,6) mutant from a T-DNA insertion screen (*282-227/Ljsym4-1*); (1,5,6) EMS-induced mutant (*EMS46/Ljsym22-1*); (5) EMS-induced mutant, T-DNA insertion mutants, somaclonal variation-induced mutants; (7,9) EMS-induced mutants (*dmi1-1/C71, dmi1-2/B129*); (8,9) fast neutron bombardment-induced mutant (*dmi1-4*)	(1) Nod- phenotype. (2) Coi- phenotype. (3,6) Nod- phenotype. No root hair curling, no IT. (3,4,6) Myc- phenotype for *Ljsym4-2*, rare infection events and delay in arbuscule formation for *Ljsym4-1* and *Ljsym22-1*. (5) No penetration of endosymbionts, with no root hairs curling neither calcium spiking during RNS, and abortion of infection attempts in AM. (7,8) Nod- phenotype.	Common SYM pathway	Cation channel that could trigger a potassium influx at the nuclear envelope and be involved in a compensatory mechanism with the release of Ca^2+^ during calcium spiking around the nucleus	(1) Szczyglowski et al., 1998 (2) Wegel et al., 1998; (3) Bonfante et al., 2000; (4) Novero et al., 2002; (5) Imaizumi-Anraku et al., 2005; (6) Kistner et al., 2005; (7) Catoira et al., 2000; (8) Ané et al., 2004
(Lj)NUP85	*L. japonicus*	Nucleoporin	Nuclear membranes	EMS-induced mutants (1,3,4,5,6) *EMS76/Ljsym24-1/Ljnup85-1*, (3,2,7) *EMS1-1E/Ljnup85-2/Ljsym73* (3,7) *EMS1-6F/Ljnup85-3/Ljsym85*	(1) Nod- phenotype for *Ljsym24*. (2) Low nodulation and Ami+. (3) Nod- and Myc- phenotypes for *Ljsym24* and *Ljsym85*; low nodulation for *Ljsym73*. (4) No ITs, low nodulation with Fix- phenotype, arbuscule formation delayed. (6) Nod- Ami- phenotypes. (5) Root hair deformation but impairment of calcium spiking. (7) Root hair branching after NF treatment, few ITs, no calcium spiking recorded, Coi- phenotype. Less AM and RNS impairments at reduced temperatures.	Common SYM pathway	NPC component	(1) Szczyglowski et al., 1998 (2) Kawaguchi et al., 2002; (3) Kawaguchi et al., 2005; (4) Kistner et al., 2005; (5) Miwa et al., 2006; (6) Sandal et al., 2006; (7) Saito et al., 2007
(Lj)NUP133	*L. japonicus*	Nucleoporin	Nuclear membranes	T-DNA insertion mutants (1,3,4) *5371-22/nup133-1/Ljsym3-1*, (1,2,4) *2557-1/nup133-2/Ljsym3-2*; (4) *nup133-4/Ljsym45*; (3,4) EMS-induced mutant *EMS247/nup133-3/Ljsym3-3*	(1) Nod- and Coi- phenotypes for *Ljsym3-2*. (2) Coi- phenotype. (3) No ITs, low nodulation with Fix- phenotype, Coi- phenotype, rarely arbuscules formation observed but strongly delayed. (4) No calcium spiking recorded for *Ljsym3-1*; for several *Ljnup133* mutants root hair swelling and branching, but not root hair curling, few ITs, strong defects in nodule development, from no nodules formed to small ineffective nodules (Fix- phenotype) with almost no infected cells. Less AM and RNS impairments at reduced temperatures.	Common SYM pathway	NPC component	(1) Schauser et al., 1998; (2) Wegel et al., 1998; (3) Kistner et al., 2005; (4) Kanamori et al., 2006
(Lj)NENA	*L. japonicus*	Nucleoporin	Nuclear membranes	EMS-induced mutants *nena-1* to *nena-5*, C6+ ion beam irradiated mutant *nena-6*	Coi- phenotype, rare successful colonization with normal arbuscules, no root hair infection, Nod- phenotype (*nena-1* and *nena-2*) to few nodules (*nena-3*), impairment of calcium spiking (*nena-1*). No symbiotic defects for *nena-4* and *nena-5*. Less AM and RNS impairments at reduced temperatures.	Common SYM pathway	NPC component proposed to be involved in import to the nucleus of proteins required for calcium spiking	Groth et al., 2010
(Mt)MCA8	*M. truncatula*	SERCA-type calcium ATPase	Nuclear membranes and ER	KD RNAi transformed roots	Silenced lines affected in NF-induced calcium spiking but no defects in nodulation, reduced AM colonization, with several aborted penetration attempts, and strongly reduced arbuscules and vesicles.	AM and RNS	Required for calcium oscillations with reloading nuclear envelope and ER lumen in calcium	Capoen et al., 2011
(Mt)SYP132	*M. truncatula*	Syntaxin	Plasma membrane, IT, unwalled droplets and symbiosome	**–**	**–**	RNS	Vesicle trafficking during IT/symbiosome formation	Catalano et al., 2004, 2007; Limpens et al., 2009; Ivanov et al., 2012
(Mt)VAMP721d and (Mt)VAMP721e	*M. truncatula*	SNARE	Vesicles closed to unwalled droplets, near or on membranes of developing symbiosomes; over PAM, in particular thin branches	KD RNAi transformed roots concerning both VAMP721d and VAMP721e	Impairment of symbiosome formation, with numerous nodular ITs but no or rare symbiosomes observed, “unwalled droplets” actually with a thin cell wall, and impairment of arbuscule formation, stopped before mature arbuscule development	AM and RNS	Common symbiotic regulators in exocytotic vesicle trafficking	Ivanov et al., 2012
(Mt)DNF1	*M. truncatula*	22-kD subunit (SPC22) of the signal peptidase complex (SPC)	ER-like structures	Fast neutron bombardment mutants	(1) Accumulation of nodule-specific cysteine-rich (NCR) peptides in the ER. (2) No terminal differentiation of bacteroids, Fix-phenotype.	RNS	Proper secretion of components involved in functional symbiosomes	(1) Van De Velde et al., 2010; (2) Wang et al., 2010b
(Ph)PAM1/(Mt)VAPYRIN	*P. hybrida*/*M. truncatula*	MSP and ANK domains	Cytoplasm, mobile spherical structures, nucleus	(1,2) Transposon mutants; (3) KD RNAi transformed roots; (4) four fast-neutron mutants, tansposon mutant lines	(1,2,3,5) Difficulties to penetrate epidermis cells, no functional arbuscules found. (4) Normal root hair curling, but numerous infection events, abnormal IT development and small, white and uninfected nodules	AM and RNS	Role in RNS and AM fungal accommodation, acting downstream of the calcium signal of the common SYM pathway, may be involved in membrane and/or cargo trafficking	(1) Sekhara Reddy et al., 2007; (2) Feddermann et al., 2010; (3) Pumplin et al., 2010; (4) Murray et al., 2011

Ami, AM infection; Coi-, Absence of cortex invasion; Fix-, defective in nitrogen fixation; KD, Knockdown; KO, Knockout; Myc-, Non-mycorrhizal; Nod-, Non-nodulating; OE, Overexpression; PGN, peptidoglycan; Pto DC3000, Pseudomonas syringae pathovar tomato strain DC3000; RNAi, RNA interference; ROS, Reactive oxygen species

* up-regulated during AM.

## Abbreviations

AM: Arbuscular mycorrhiza
CCaMK: Calcium and calmodulin-dependent protein kinase
DMI: Does not make infections
ER: Endoplasmic reticulum
IT: Infection thread
LCO: Lipochitooligosaccharide
LRR: Leucine-rich repeat
LYK: LysM-containing receptor-like kinase
LYM: LysM-containing protein
LysM: Lysin motif
MAPK: Mitogen-activated protein kinase
MF: Myc factor, mycorrhization factor
MFR: Myc factor receptor
NCR: Nodule-specific cysteine-rich
NF: Nod factor, nodulation factor
NFR: Nod factor receptor
NPC: Nuclear pore complex
PAM: Periarbuscular membrane
PIT: Pre-infection thread
PPA: Prepenetration apparatus
PT: Phosphate transporter
RNS: Root nodule symbiosis
SNARE: soluble *N*-ethylmaleimide-sensitive factor attachment protein receptor
SPC: Signal peptidase complex
SYMRK: Symbiosis receptor kinase
SYP: Syntaxin of plants
VAMP: Vesicle-associated membrane protein.
